# „Bottoms-up“ portal venous recanalization TIPS (PVR-TIPS) utilizing a re-entry catheter

**DOI:** 10.1186/s42155-024-00510-1

**Published:** 2024-12-23

**Authors:** Alexander Loizides, Martin Freund, Heinz Zoller, Benedikt Schäfer

**Affiliations:** 1 Department of Radiology, Medical University Innsbruck, Anichstrasse 35, Innsbruck, 6020 Austria; 2 Department of Gastroenterology, Medical University Innsbruck, Anichstrasse 35, Innsbruck, 6020 Austria

**Keywords:** PVR-TIPS, Portal vein thrombosis, TIPS, Re-entry catheter

## Abstract

**Background:**

Three patients with portal hypertension and gastrointestinal bleeding due to non-cirrhotic portal vein thrombosis were treated with portal venous recanalization transjugular intrahepatic portosystemic shunt (PVR-TIPS) via a trans-splenic access.

**Main body:**

A “bottoms-up” retrograde puncture of the right hepatic vein was performed using a re-entry catheter to gain access to the right hepatic vein. In all patients a successful retrograde puncture of the right hepatic vein was achieved, thereby restoring the splenoportal tract.

**Conclusion:**

Our cases present an alternative approach to treat chronic portal vein thrombosis expanding the possibilities of the PVR-TIPS procedure.

## Background

Recent guidelines recommend portal vein recanalization (PVR) followed by a transjugular intrahepatic portosystemic shunt (TIPS) in patients with chronic portal vein thrombosis and recurrent bleeding not manageable medically or endoscopically [1]. Current knowledge about the fairly novel procedure is based on small retrospective studies and case series [2, 3]. The transsplenic method for reaching the thrombosed portal vein appears to outperform the transhepatic approach [4]. PVR-TIPS studies have focused on cirrhotic patients, mainly liver transplant candidates, aiming to facilitate a natural connection between the graft and the recipient’s portal vein [5]. Here we report an alternative approach for portal venous recanalization in three patients with non-cirrhotic portal vein thrombosis.

## Main body

### Case descriptions

#### Case 1

A 29-year-old woman was admitted with progressive fatigue and melena. The hemoglobin concentration at admission was 4.5 g/dL. Endoscopy showed large oesophageal varices with red spots and prophylactic band ligation was performed. Magnetic resonance imaging revealed a portal vein thrombosis with cavernous transformation as well as splenomegaly. Platelet count was normal (160 G/L), despite portal hypertension. Molecular testing for the V617F mutation in JAK2 was positive and bone marrow testing confirmed a myeloproliferative neoplasm, compatible with the subtype essential thrombocythemia. Treatment with the JAK2 inhibitor ruxolitinib, carvedilol 6.25 mg b.i.d. and anticoagulation with apixaban 5 mg b.i.d. was initiated. Ongoing, portal hypertensive bleeding, anemia and insufficient regression of esophageal varices represented the indication for a portal vein recanalization procedure.

#### Case 2

A 49-year old man with a history of chronic pancreatitis presented with a 6 cm pseudo-aneurysm of the inferior pancreaticoduodenal artery, which compressed the portal vein and caused total thrombotic occlusion and cavernous transformation. The aneurysm was treated with embolization but persistent portal hypertension was evident with large esophageal and fundal varices. The patient also developed portal biliopathy complicated by recurrent cholangitis, necessitating repeated endoscopic biliary drainage. Additionally a transfusion-dependent anemia due to gastrointestinal bleeding was developed, before being transferred to our centre for portal vein recanalization.

#### Case 3

A 64-year old woman presented with abdominal pain, nausea and vomiting. Ultrasound and CT imaging revealed a complete portal and vein thrombosis which extended to the superior mesenterial vein. Prefibrotic primary myelofibrosis (MPL W515L positive) was identified as a likely cause for portal vein thrombosis. Endoscopy showed small esophageal varices and hypertensive gastropathy with extensive bleeding which provided the indication for portal vein recanalization.

### Procedure

**Fig. 1:**
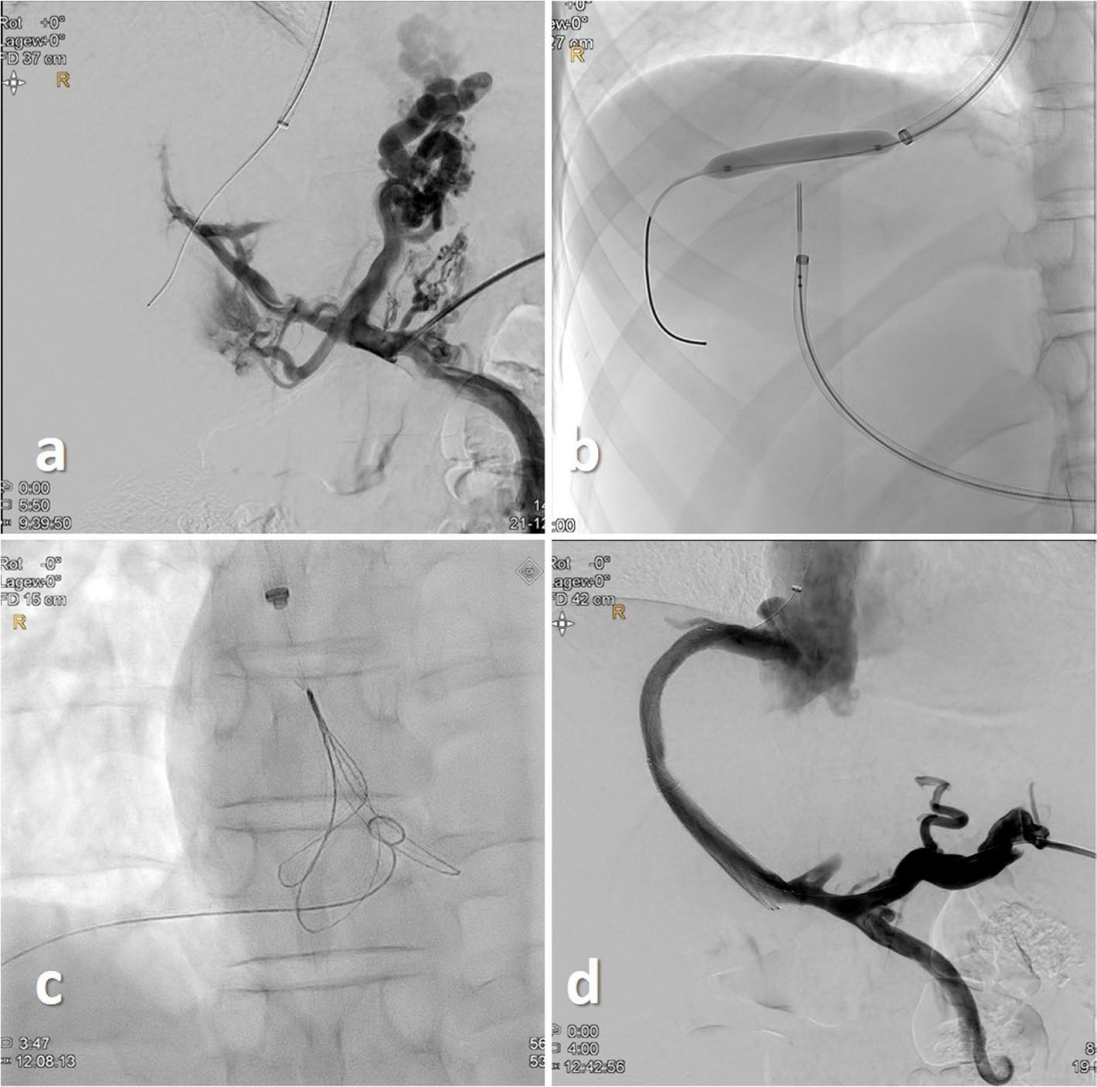
Case 1-DSA via a transsplenic access depicting the subtotal thrombosed PV as well as large gastrooesophageal varices **a**. „Bottoms-up“ retrograde puncture of an inflated balloon in the RHV using a re-entry catheter system **b**. Snaring of a 0.018 inch guidewire in the right atrium **c**. Final DSA depicting a patent splenoportal tract **d**

**Fig. 2:**
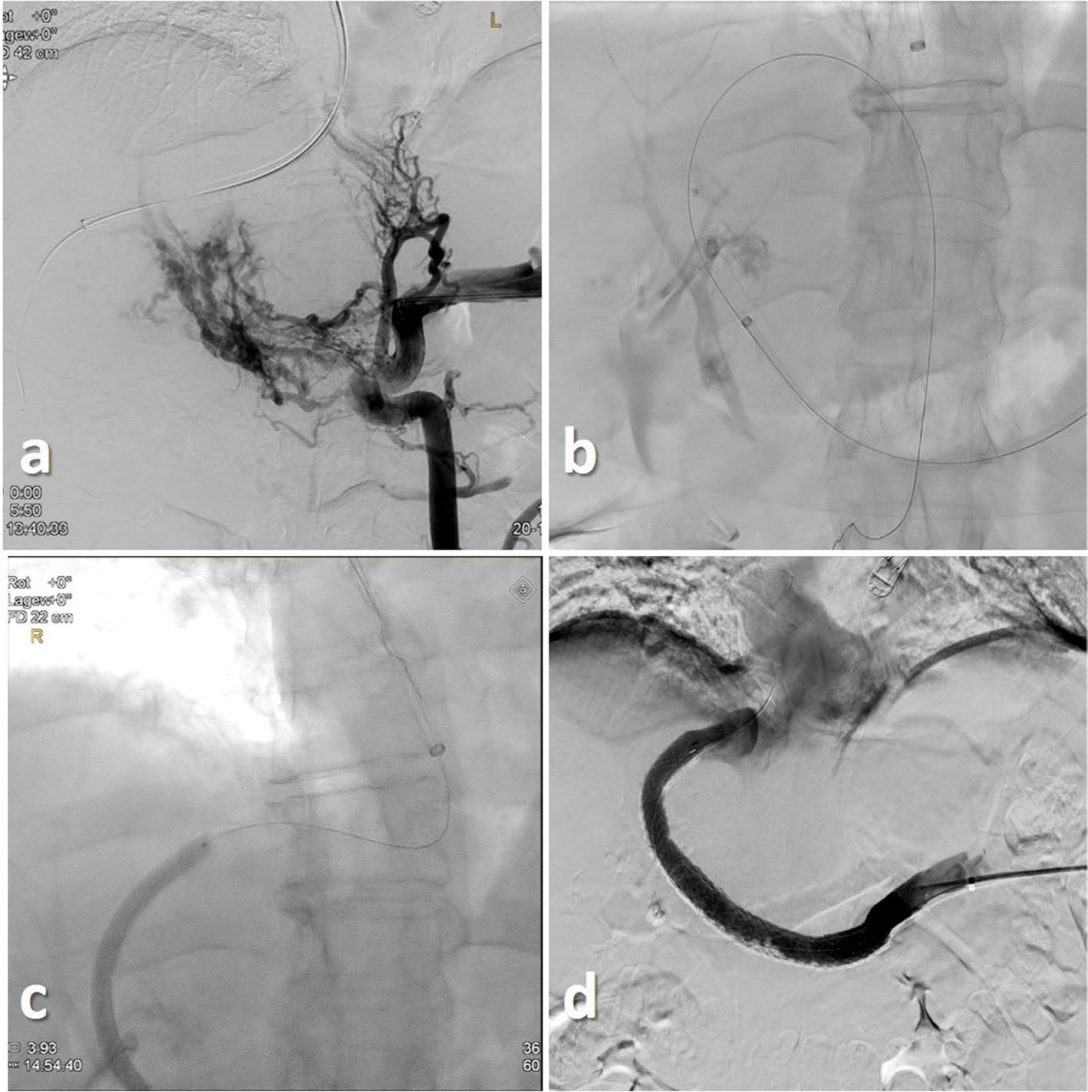
Case 2-DSA via a transsplenic access depicting a cavernous transformation of the PV as well as gastrooesophageal varices **a**. After „Bottoms-up“ retrograde direct puncture of the RHV a 0.018 inch guidewire was advanced in the IVC **b**. Snaring of the guidewire in the right atrium **c**. Final DSA depicting a patent splenoportal tract **d**

**Fig. 3:**
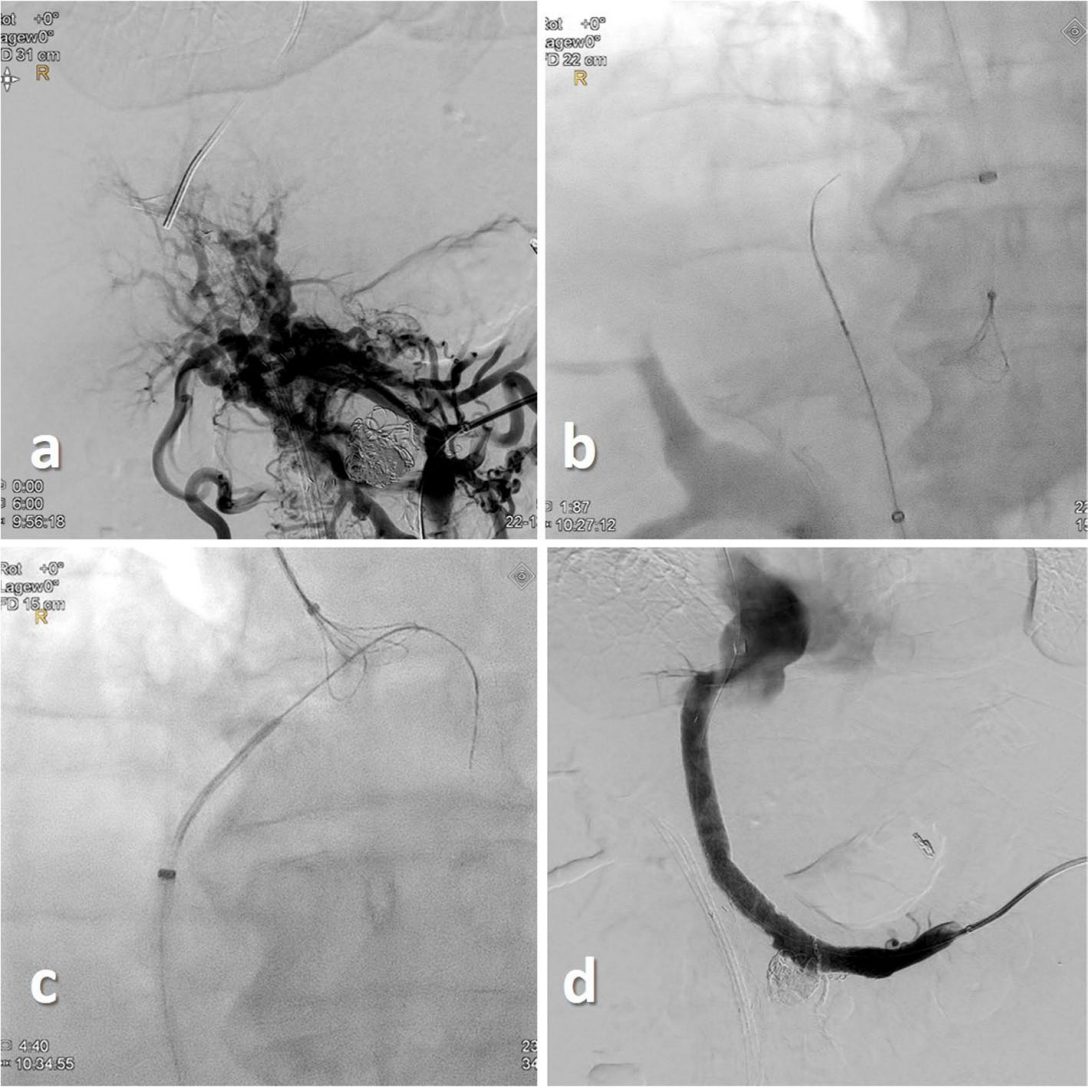
Case 3 - DSA via a transsplenic access cavernous transformation of the PV **a**. „Bottoms-up“ retrograde direct puncture of the RHV using a re-entry catheter system **b**. Snaring of a 0.018 inch guidewire in the right atrium **c**. Final DSA depicting a patent splenoportal tract **d**

All procedures were performed in general anesthesia and cardiovascular monitoring. Under sterile conditions, the right jugular vein was accessed under ultrasound guidance in seldinger-technique using a micropuncture 21G needle set (Cook Medical, Bloomington, IN, US). After predilation of the access tract with 10 F and 12 F dilatators a 10 F (45 cm) sheath (Cook Medical, Bloomington, IN, US) was introduced. Subsequently the right atrial and pulmonary artery pressure were measured using a 145° angulated Pigtail catheter (Cordis, Miami Lakes, FL, US). After that, a 5 F headhunter catheter (Cordis, Miami Lakes, FL, US) was positioned in the right hepatic vein. Next, an ultrasound-guided transsplenic approach was used to gain access to a central intraparenchymal perihilar branch of the splenic vein (SV) using a micropuncture needle set. Initially, a 6 F KCFW sheath (Cook Medical, Bloomington, IN, US) was introduced before subsequently a diagnostic catheter portography was performed revealing a regular distal SV but a subtotal occlusion or cavernous transformation of a thin appearing portal vein (PV) as well as large paragastral and paraesophageal varices. Following a recanalization of the thrombotic/postthrombotic PV was achieved using a hydrophilic guidewire (Terumo Corporation, Tokyo, Japan) and a 5 F multipurpose catheter (Terumo Corporation, Tokyo, Japan). A contrast-filled high-pressure 6/40 balloon (Powerflex Pro, Cordis, Miami Lakes, FL, US) was positioned in an intrahepatic right PV. A “bottoms-up” retrograde puncture of the right hepatic vein was performed using a re-entry catheter to gain access to the right hepatic vein: In case 1 a contrast-filled high-pressure 6/40 balloon was positioned in the RHV and a “bottoms-up” retrograde puncture was performed using a re-entry catheter device (BeBack Crossing Catheter, Bentley InnoMed GmbH, Hechingen, Germany) which was positioned in the predilated right PV. In case 2 and case 3 a direct puncture of the RHV was performed using again a re-entry catheter device. Thereby a successful puncture of the RHV could be achieved in all three patients. Thereafter a 0.018 inch guidewire could be advanced through the RHV in the inferior vena cava (IVC) or right atrium, respectively. Using the transjugular access a 18–30 mm 3D Snare System (EN Snare Endovascular Snare System, Merit, South Jordan, US) was advanced into the right atrium and the 0.018 inch guidewire was subsequently snared to establish a transjugular-transsplenic pull-through wire. Using this access a TIPS-Stentgraft (8–10/8 + 2 Viatorr, W. L. Gore & Associates Inc, Newark, DE, US) was introduced after predilation of the access-tract initially with a 4/100 low-profile balloon and a high-pressure 6/60 balloon. In case 1, a 14/80 non-covered self-expandable Nitinol-stent (S.M.A.R.T. Control, Cordis, Miami Lakes, FL, US) was positioned between the TIPS-Stentgraft and the “healthy”, non-thrombotic proximal PV respectively the venous confluence and dilated using a high-pressure 12/40 balloon achieving a recanalization of the splenoportal tract. In case 2 and case 3 a 14/100 Beyond Venous Stent (Bentley InnoMed GmbH, Hechingen, Germany) was used, extended with an additional 14/80 Beyond Venous Stent to the proximal SV and dilated using a high-pressure 12/40 balloon (Figs. 1, 2 and 3).

Anticoagulation with unfractionated heparin was initiated during the procedure and then switched to low molecular weight heparin to prevent thrombotic stent occlusion in all three patients. All three patients received broad-spectrum antibiotics. Low molecular heparin was switched to apixaban for long-term anticoagulation.

Two years post-intervention, Case 1 demonstrated excellent overall health, with only minor residual varices and no further episodes of gastrointestinal bleeding. In contrast, Case 2 required hospital readmission two months after the PVR-TIPS procedure due to bleeding from the gastroduodenal and inferior pancreaticoduodenal arteries. This complication was successfully managed with coil embolization and was likely attributable to the patient’s underlying chronic pancreatitis. At the one-year follow-up, no additional bleeding episodes were observed, and the patient remained in stable condition. Similarly, Case 3 experienced an uneventful one-year follow-up, with no further bleeding incidents reported.

## Discussion

Hypercoagulable conditions and reduced portal blood flow are major risk factors for the development of portal vein thrombosis which mostly occurs in individuals with cirrhosis. Non-cirrhotic portal hypertension is a rare condition usually found in patients with acquired hematological disorders or inherited thrombophilia [1]. Ensuing compensatory mechanisms create venous collaterals to bypass the obstructed portal vein. This not only leads to varices but the resulting cavernous transformation of the portal vein itself poses additional risks like biliary obstruction [1, 6]. The PVR-TIPS procedure has emerged as a pivotal intervention in managing portal vein thrombosis and is mainly used in cirrhotic candidates for liver transplantation as described by Riad S. and colleagues [7, 8]. By recanalizing the thrombosed portal vein and simultaneously creating a portosystemic shunt, the procedure effectively alleviates portal hypertension and restores portal venous flow and thereby alleviates the risk for bleeding as well as other complications and offers a treatment option for refractory ascites [9–11]. It can also improve surgical aspects of portal vein anastomosis in patients receiving a liver transplantation where alternative portal vein anastomoses are associated with poorer outcome [6, 12]. Careful consideration of the potential risks and benefits of the often challenging procedure is necessary. Risks associated with the procedure include bleeding, infection, thrombosis and injury to the liver, spleen or other organs. In some cases, the shunt can also narrow or occlude, which may require further intervention. Lifelong anticoagulation may be necessary to avoid thrombosis of the stent graft. In our three patients, the decision to implement lifelong anticoagulation was based on the prothrombotic state associated with their underlying conditions. Currently, there are no clear recommendations for or against anticoagulation after PVR-TIPS.

Although the conventional transjugular puncture is preferred in a TIPS procedure, this “bottoms-up” retrograde puncture should be considered in challenging cases as an alternative approach, showing certain advantages as illustrated in our case series: the conventional puncture technique aims the right portal vein, hence one vessel. In contrary, a retrograde bottoms-up puncture aims at one of the RHV side branches which subsequently lead to the RHV. We hypothesize that a wider puncture area using a bottoms-up approach could enable an easier access to the RHV. Additionally, accidental transcapsular perforation can be avoided using a “bottoms-up” approach.

## Conclusion

Our cases present an alternative approach to treat chronic portal vein thrombosis with recurrent bleeding which could not be managed medically or endoscopically, expanding the possibilities of the PVR-TIPS procedure.

## Data Availability

All data generated or analysed during this study are included in this published article.
